# Paraneoplastic acquired ichthyosis as the first manifestation in breast implant-associated anaplastic large cell lymphoma^⋆^

**DOI:** 10.1016/j.abd.2022.12.009

**Published:** 2024-04-06

**Authors:** Héctor Chiang Wong, Pilar Martínez Sánchez, María del Carmen González Guzmán

**Affiliations:** a Hematology Unit, José Carrasco Arteaga Hospital, Cuenca, Ecuador; b Hematology Unit, 12 de Octubre Hospital, Madrid, España; c Rheumatology Unit, José Carrasco Arteaga Hospital, Cuenca, Ecuador

Dear Editor,

Anaplastic large cell lymphoma (ALCL) accounts for 1%–3% of non-Hodgkin's lymphomas and approximately 15% of T-lymphomas. The 5th edition of the World Health Organization (WHO) classification of hematolymphoid tumors recognizes four different subtypes of ALCL: ALK-positive, ALK-negative, primary cutaneous, and those associated with breast implants (BIA-ALCL).

In January 2011, the Food and Drug Administration (FDA) published 34 cases collected between 1997 and 2010, with a probable association between ALCL and the use of breast implants. Owing to the implementation of the PROFILE registry, approximately 900 cases have been reported worldwide.

Implants are categorized according to their internal fill (saline or silicone), shape (symmetric or asymmetric), or external surface (smooth or textured).^1^, ^2^ Textured implants have the greatest association with this type of lymphoma.

We present the case of a 43-year-old woman with a history of bilateral breast prosthesis implantation (silicone-textured), 6-years ago for aesthetic reasons. She visited the clinic because of the presence of scaly plaques with cracks and erythematous edges throughout her body, with a 3-month history. In some plaques, fine superficial scaling was prominent in the intermammary fold, scalp, and on the edge of the eyelids. Physical examination revealed a fixed, painless, 1.5 cm adenopathy in the right axillary region (Fig. 1).

**Fig. 1:**
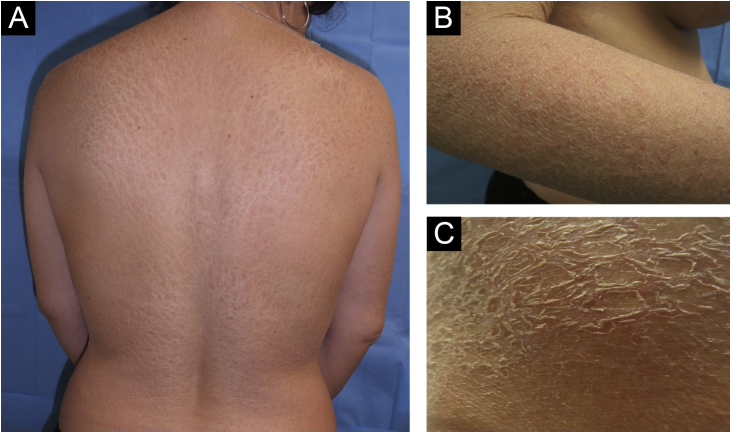
(A, B and C) scaly plaques with cracks and erythematous edges (ichthyosis).

Chest CT scan, along with a whole-body PET/CT, revealed a liquid collection surrounding the right mammary implant, with an increase in soft tissues in the chondrosternal joint, poor definition of the pectoral planes in its medial portion, and trabeculation of subcutaneous fat. In contrast, multiple pathological adenopathies were observed in the anterior mediastinum, bilateral axillary regions, and supraclavicular fossae (Fig. 2A‒C).

**Fig. 2:**
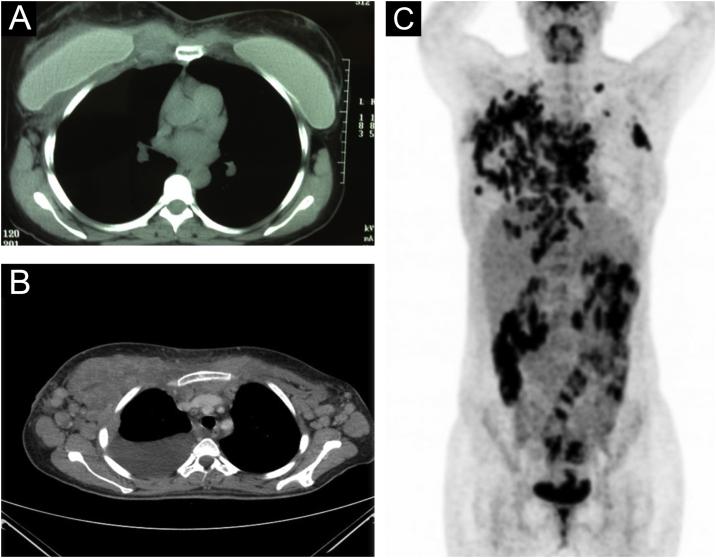
(A‒B) Liquid collection surrounding the right mammary implant, with an increase in soft tissues in the chondrosternal joint, poor definition of the pectoral planes in its medial portion, and trabeculation of subcutaneous fat. (C) Multiple pathological adenopathies were observed in the anterior mediastinum, and bilateral axillary regions and supraclavicular fossae.

While a skin biopsy was suggestive of ichthyosis, subsequent biopsy of the right axillary lymph node revealed neoplastic cells with a multilobulated anaplastic morphology and an “embryoid” appearance with foci of tumor necrosis. Immunohistochemistry phenotypes were CD45+ CD30+, CD43+, Bcl-2+, MUM-1+, MIB-1+(60%), CD3-, CD20-, CD79a-, CD10-, BCL6-, CD38-, LMP1-, ALK-, suggestive of anaplastic large cell lymphoma (Fig. 3A‒F).

**Fig. 3:**
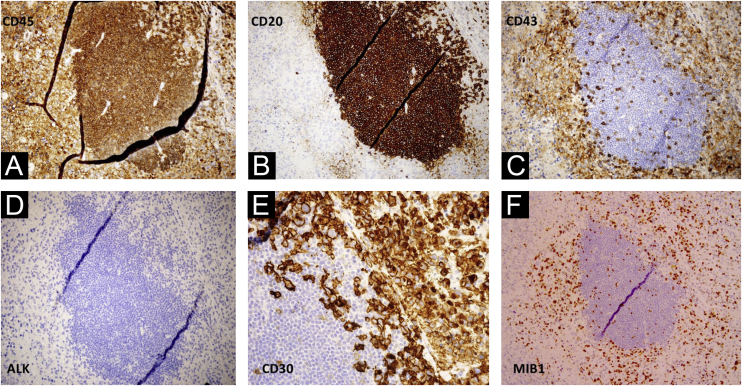
(A‒F) Biopsy of the right axillary lymph node: CD45+ CD30+, CD43+, Bcl-2+, MUM-1+, MIB-1+(60%), CD3-, CD20-, CD79a-, CD10-, BCL6-, CD38-, LMP1, ALK-, suggestive of anaplastic large cell lymphoma.

Based on the clinical, radiological, and pathological findings, the diagnosis of BIA-ALCL with a locally aggressive or extensive stage was established (stage III, T4N2M0).

BIA-ALCL commonly presents as a late peri-implant seroma (>80%), causing distortion and asymmetry of the breasts. It can manifest with regional lymphadenopathy (predominantly axillary, supra-, or infra-clavicular) in 20% of cases, or concurrent with skin lesions (erythema, rash, erythematous nodules) or as de novo in 8% of cases.^3^, ^4^, ^5^, ^6^, ^7^

In our case, ichthyosis was the initial cutaneous paraneoplastic manifestation of BIA-ALCL and dermatological management was based on glycerin soap, liquid petroleum jelly, and methylprednisolone aceponate (ointment). This cutaneous alteration can appear in the same manner in endocrinopathies, immunological disorders, vitamin deficiencies, infections, and with drug use.^3^

BIA-ALCL originates from the fibrous capsule surrounding the implant, except in the advanced stages, which involve the surrounding tissue. Stage I was diagnosed in 83% of patients, and only 7% presented with stage IV disease.^1^, ^6^

The etiopathogenesis of BIA-ALCL is multifactorial, namely chronic infection in the textured implant configuration, gram-negative biofilm formation, immune response, chronic inflammation, host genetics (e.g., JAK/STAT, p53), and timing of oncogenesis. The presumptive triggers include mechanical friction, silicone implant shell particles, silicone leachate, and bacteria. BIA-ALCL presents at a median time of 8–10 years after breast prosthetic implantation.^6^, ^7^, ^8^, ^9^

Considering that 2%–4% of cases can occur bilaterally, both implants of our patient were removed with total capsulectomy,^1^, ^6^, ^8^, ^10^ and chemotherapy was initiated under the CHOP regimen (cyclophosphamide, doxorubicin hydrochloride, vincristine sulfate, and prednisone). After six cycles, cthe complete morpho-metabolic response of the patient was confirmed by PET/CT, with subsequent disappearance of the skin lesions.

We must establish the role of certain agents (brentuximab vedotin), adjuvant therapies (radiotherapy), and autologous stem cell transplantation, mainly in locally aggressive or advanced stages (stages II‒IV).

In conclusion, the discussed case of a patient with BIA-ALCL who presented with acquired ichthyosis, emphasizes a multidisciplinary approach for early diagnosis and corresponding management.

## Financial support

None declared.

## Authors’ contributions

Héctor Chiang Wong: Approval of the final version of the manuscript, critical literature review, data collection, analysis and interpretation; intellectual participation in propaedeutic and/or therapeutic management of the studied case.

Pilar Martínez Sánchez: Approval of the final version of the manuscript, data collection, intellectual participation in propaedeutic and/or therapeutic management of studied case.

María del Carmen González Guzmán: Approval of the final version of the manuscript, data collection, analysis, and interpretation.

## Conflicts of interest

None declared.
